# Association Between Preeclampsia Risk and Fine Air Pollutants and Acidic Gases: A Cohort Analysis in Taiwan

**DOI:** 10.3389/fpubh.2021.617521

**Published:** 2021-03-31

**Authors:** Shih-Yi Lin, Yu-Cih Yang, Jun-Wei Su, Jie-Sian Wang, Chang-Cheng Jiang, Chung-Y. Hsu, Chia-Hung Kao

**Affiliations:** ^1^College of Medicine, Graduate Institute of Biomedical Sciences and School of Medicine, China Medical University, Taichung, Taiwan; ^2^Division of Nephrology and Kidney Institute, China Medical University Hospital, Taichung, Taiwan; ^3^Management Office for Health Data, China Medical University Hospital, Taichung, Taiwan; ^4^College of Medicine, China Medical University, Taichung, Taiwan; ^5^Department of Gynecology, China Medical University Hospital, Taichung, Taiwan; ^6^Department of Nuclear Medicine and PET Center, China Medical University Hospital, Taichung, Taiwan; ^7^Department of Bioinformatics and Medical Engineering, Asia University, Taichung, Taiwan; ^8^Center of Augmented Intelligence in Healthcare, China Medical University Hospital, Taichung, Taiwan

**Keywords:** air pollutant, preeclampsia risk, national health insurance research database, Taiwan air quality monitoring database, risk assessment

## Abstract

**Background:** Fine air pollutant particles have been reported to be associated with risk of preeclampsia. The association between air pollutant exposure and preeclampsia risk in heavily air polluted Taiwan warrants investigation.

**Methods:** We combined data from Taiwan National Health Insurance (NHI) Research Database (NHIRD) and Taiwan Air Quality Monitoring Database. Women aged 16–55 years were followed from January 1, 2000, until appearance of ICD-9 coding of preeclampsia withdrawal from the NHI program, or December 31, 2013. Daily concentration of NOx, NO, NO_2_, and CO was calculated by Kriging method. The Cox proportional hazard regression model was used for risk assessment.

**Results:** For NOx, Relative to Quartile [Q] 1 concentrations, the Q2 (adjusted hazard ratio adjusted = 2.20, 95% CI = 1.50–3.22), Q3 (aHR = 7.28, 95% CI = 4.78–11.0), and Q4 (aHR = 23.7, 95% CI = 13.7–41.1) concentrations were associated with a significantly higher preeclampsia or eclampsia risk. Similarly, for NO, relative to Q1 concentrations, the Q2 (aHR = 1.82, 95% CI = 1.26–2.63), Q3 (aHR = 7.53, 95% CI = 5.12–11.0), and Q4 (aHR = 11.1, 95% CI = 6.72–18.3) concentrations were correlated with significantly higher preeclampsia or eclampsia risk. Furthermore, for NO_2_, relative to Q1 concentration, the Q2 (aHR = 1.99, 95% CI = 1.37–2.90), Q3 (aHR = 6.15, 95% CI = 3.95–9.57), and Q4 (aHR = 32.7, 95% CI = 19.7–54.3) concentrations also associated with a significantly higher preeclampsia or eclampsia risk.

**Conclusion:** Women exposed to higher NO_X_, NO, NO_2_, and CO concentrations demonstrated higher preeclampsia incidence.

## Introduction

Preeclampsia, a multiorgan syndrome manifested as hypertension and proteinuria, occurs in pregnant woman after 20 weeks of gestation (1). It affects 2–8% of all pregnancies and remains a leading cause of maternal morbidity and mortality worldwide (2). Early- or late-onset of preeclampsia is associated with high respiratory morbidity, cardiomyopathy, cardiovascular morbidity, and acute renal failure rates (3). Furthermore, large cohort studies have demonstrated that gestational hypertensive women had increased risk of developing systemic lupus erythematosus (4), intracranial hemorrhage (4), and end-stage renal disease (5) later in life.

In addition to its effects on maternal health, preeclampsia may also affect fetal well-being (6): preeclampsia may increase incidence of stillbirth, intrauterine growth restriction, bronchopulmonary dysplasia, and transient hematological disorder (7), and preeclamptic pregnancies could lead to increased risk of neonatal death (6). A cohort study including one million children reported children exposed to preeclampsia as a fetus could have an increased risk of endocrine, nutritional, and metabolic derangements in their adolescence and early adulthood (8). Taken together, preeclampsia can have long-term effects in the affected mothers and children.

The currently recognized risk factors for preeclampsia are high body mass index, hypertension or family history of hypertension, ethnicity, smoking, nulliparity, work during pregnancy, antiphospholipid syndrome, multiple pregnancies, insulin-dependent diabetes, and age > 40 years (5–7). Beyond the individual risk factors, whether environmental pollutants influence preeclampsia risk has aroused interest: increased preeclampsia risk has been linked to volatile organic compound (8) and fine particulate air pollution (9) exposure. Moreover, a Chinese study reported that prenatal exposure to PM_10_ and SO_2_ could increase preeclampsia risk (10). However, similar data about preeclampsia risk and air pollutant exposure in Taiwan is lacking. Due to the geographic location of Taiwan, most air pollutants are imported from China with northeast monsoons each winter (11). In addition, Taiwan has the largest coal combustion-based thermal power plant in the world, which supplies ~4,400 MW of electricity to mid-Taiwan by coal burning (12). In Taiwan, the average concentration of particulate matter with aerodynamic diameter <2.5 μm (PM_2.5_) is 42.8 μg/m^3^ (13), compared with concentrations of merely 14.0 ± 0.22 μg/m^3^ in the United States (14). Therefore, an investigation of the association between air pollutant exposure and preeclampsia in the heavily polluted Taiwan is warranted. We combined information from Taiwan National Health Insurance Research Database (NHIRD) and Taiwan Air Quality Monitoring Database (TAQMD) to investigate whether preeclampsia risk increased with increasing concentrations of air pollutants.

## Methods

### Data Source

The data used in the present study were sourced from the Longitudinal Health Insurance Database 2000 (LHID2000). LHID2000 is a representative subset of data that includes data from 1,000,000 individuals systematically and randomly selected from the year 2000 registry of beneficiaries of the Taiwan National Health Insurance Research Database (NHIRD). The LHID 2000 contained the comprehensive de-identified healthcare information on demographic, outpatient visit, inpatient care, prescription drugs and medical procedures from 1996 to 2013. The NHIR confirmed that there no significant differences in the distribution of age, sex, or health care costs between the population in the LHID 2000 and those in the NHIRD. This study uses the International Classification of Disease, Ninth Revision, Clinical Modification (ICD-9-CM) to categorize disease diagnoses based on inpatient data.

The air pollution data were retrieved from Taiwan Air Quality-Monitoring Database (TAQMD), which has been gathering the information from 78 air-quality monitoring stations in Taiwan since 1993. Most monitoring stations are located in populous urban and rural areas and can be used to inform the public on ambient air quality. The locations of the monitoring stations and air pollution sources were identified and managed by geographic information system (GIS) (ArcGIS version 10; ESRI, Redlands, CA, USA) (15). Open data on hourly measurements of major pollutants are available on the monitoring network website. These hourly data recorded at each TAQMD were further averaged into daily mean concentrations in this study. The air pollutant monitoring stations routinely monitor several pollutant levels, including Nitrogen oxides, Carbon oxides, and particulate matter concentrations, as well as weather condition, such as temperature and humidity (Supplementary Figure 1) (16).

### Ethics Statement

The NHIRD encrypts patient personal information to protect privacy and provides researchers with anonymous identification numbers associated with relevant claims information, including sex, date of birth, medical services received, and prescriptions. Therefore, patient consent is not required to access the NHIRD. This study was approved to fulfill the condition for exemption by the Institutional Review Board (IRB) of China Medical University (CMUH-104-REC2-115-AR4). The IRB also specifically waived the consent requirement.

### Sample Participant

**Figure 1:**
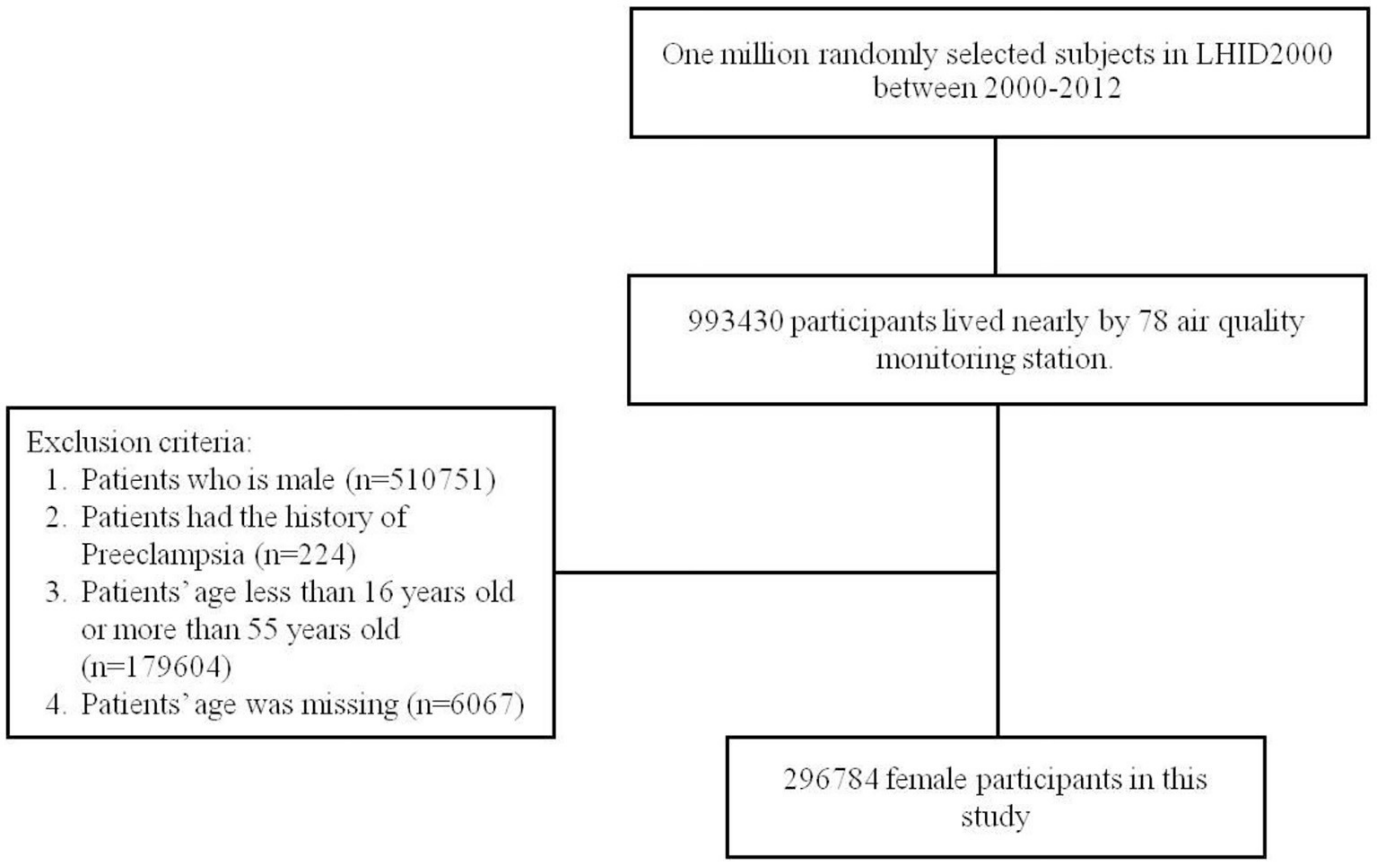
Flow chart of this study.

People residing in the area where an air quality-monitoring station was located as the population. People with a history of pre-eclampsia and eclampsia (ICD-9-CM code 642.4, 642.5, 642.6, and 642.7) before 2000 were excluded. All participants were contained 16–55 years and followed from January 1, 2000 until the diagnosis of preeclampsia withdrawal from the NHI, or December 31, 2013. We have enrolled certain confounding factors as possible which have been reported to be risk factors of preeclampsia (7, 17–21) which included age, urbanization level of residence, monthly income, occupational class, and comorbidities such as hypertension (ICD-9-CM 401-405), diabetes mellitus (ICD-9-CM 250), chronic kidney disease (ICD-9-CM 580-589), obesity (ICD-9-CM 278), glomrulonephritis (ICD-9-CM 582.1), multiple pregnancy (ICD-9-CM 72-74, 650-659, and 640-676), autoimmune disease (ICD-9-CM 710.0-710.4, 714.0, 714.30-714.33, 340, 446.0-446.2, 446.4-446.5, 446.7, 443.1, 136.1, 694.4, 555, 556.0-556.6, and 56.8-556.9), nephrotic syndrome (ICD-9-CM 581.9), proteinuria (ICD-9-CM 791.0), previous Preeclampsia (ICD-9-CM 642.7), and Multiple births (ICD-9-CM 651, 652.6, 660.5, 662.3, and v27.2-v27.7). The NHRI stratified all city districts and townships in Taiwan into 7 urbanization levels, based on population density (people/km^2^), proportion of residents with higher education, elderly and agricultural population, and the number of physicians per 100,000 people in each area. Level 1 represented areas with a higher population density and socioeconomic status, and level 7 represented the lowest. Because few people lived in more rural areas of levels 4-7, our study grouped these areas into the level 4 group. Monthly income was classified into 3 groups: < NT$15,000, NT$15,000–29,999, and >NT$30,000. The occupational class was divided to three classes, white collar, blue collar, and other class. The white collar people who work in offices doing work that needs mental rather than physical effort. The blue collar workers do work needing strength or physical skill rather than office work such as farmer or fisherman. The other classes contain unemployed people, soldier, and religious people (Figure 1). As for the validity of diagnosed validity of “preeclampsia” in the NHIRD, the Ministry of Health and Welfare of Taiwan recently initiated a national validation project using existing registry data to improve the accuracy of the NHIRD (22, 23). In addition, previous many studies have verified the completeness and showed high levels of validity of coding “preeclampsia” in Taiwan's NHIRD (24–30).

### Outcome and Exposure Measurement

The end point in this data is pre-eclampsia and eclampsia (ICD-9-CM code 642.4, 642.5, 642.6, and 642.7). We obtained hourly data of NOx, NO, NO_2_, and CO from 78 monitoring stations constructed by Taiwan Environmental Protection Administration (TEPA) on Taiwan's main island that provided measurement continuously from 1999 to 2013. Methods used for measuring these pollutants were chemiluminescence for NOx, NO, NO_2_, and non-dispersive infrared absorption for CO. These data were subjected to rigorous quality assurance and control procedures through independent projected. TEPA authorized an independent private sector to perform annual performance audits and regular performance checks for monitoring instruments. The information of the patients located in the ArcGIS system was taken from the location of the administrative agency corresponding to the postal code of the patient's registered residence in the NHIRD. The postal code typically corresponded to one block face in urban areas (mean ± SD: 17 ± 8.56 square kilometer) but was larger in rural areas (mean ± SD: 154 ± 104.39 square kilometer) with low population density (15). We use the latitude and longitude coordinates of the location of the relevant administrative agency to input into the ArcGIS system to locate each patient in the research group.

To estimate the effect of air pollutants on pre-eclampsia and eclampsia, we speculate the daily concentration of NOx, NO, NO_2_, and CO by Kriging method. Kriging models are originated in the areas of mining and geostatistics that involve spatially and temporally correlated data. Their unique characteristic stems from their ability to combine global and local modeling. Kriging is a multistep process; it includes exploratory statistical analysis of the data, variogram modeling, creating the surface, and (optionally) exploring a variance surface. Kriging predicts values of unknown points based on the distance of direction between sample point reflects a spatial correlation that can be used to explain variation in the surface. It fits a mathematical function to a specified number of points, or all point within a specified radius, to determine the output value for each location. Under suitable assumptions on the priors, kriging gives the best linear unbiased prediction of the intermediate values. The value estimated by the Kriging method will be more accurate. The air pollutant exposure levels were scaled to the interquartile range (IQR) in this study.

Air pollutants were also categorized into quartiles with three cut-off point (25, 50, and 75th percentiles) for NOx (Quartile 1, <19.8 ppb; Quartile 2, 19.8–25.6 ppb; Quartile 3, 25.6–35.9 ppb; Quartile 4, >35.9 ppb), NO (Quartile 1, <4.24 ppb; Quartile 2, 4.24–5.98 ppb; Quartile 3, 5.98–12.6 ppb; Quartile 4, >12.6 ppb), NO_2_ (Quartile 1, <15.6 ppb; Quartile 2, 15.6–19.6 ppb; Quartile 3, 19.6–23.6 ppb; Quartile 4, >23.6 ppb), and CO (Quartile 1, <0.43 ppm; Quartile 2, 0.43–0.51 ppm; Quartile 3, 0.51–0.61 ppm; Quartile 4, > 0.61 ppm).

### Statistical Analysis

The chi-squared test was used to compare the differences of urbanization level in each quartiles of daily average concentration of air pollutants. We conducted Cox-proportional hazards regression for multivariable analysis of the diagnosis of preeclampsia by IQR increase (μg/m^3^, ppb or ppm) of long-term exposure to SO_2_, NOx, NO, NO_2_, and CO, from 2000 to developing pre-eclampsia and eclampsia or the end of the study period. The incidence of pre-eclampsia and eclampsia (per 10,000 person-years) was calculated at each levels of air pollutant concentrations. The relative risk of pre-eclampsia and eclampsia in participants who exposed under Q2-Q4 level of air pollutant concentrations corresponding to those who exposed in Q1 level of air pollutant concentrations was estimated by Cox proportional hazard regression model. The potential confounders which contains age, monthly income, urbanization level, occupational class, and comorbidities were defined as determinants of pre-eclampsia and eclampsia and associated with air pollution concentrations and further incorporated into models. We also preformed Cox regression model which stratified by age, urbanization level, monthly income and occupational class for analyzing risk of pre-eclampsia and eclampsia between yearly average concentrations of air pollutants.

## Results

Table 1 shows that the baseline characteristics and air pollutant exposure of the study cohort. Among the 296,784 female subjects, insured persons in 30–44 years of age (41.8%), living in north area (48.0%), living in urbanization level 1 (33.4%), monthly income in 15,000–29,999 (47.3%), and white collar class worker (57.9%) were dominant. The mean age of the study subjects of the study subjects were 33.7 (± 10.4) years old. The mean follow up time was 11.9 (± 0.25) years. The mean of yearly air pollutants concentration were 29.2 (± 12.8) ppb for NOx, 9.47 (± 8.18) ppb for NO, 19.7 (± 5.42) ppb for NO_2_, and 0.55 (± 0.18) ppm for CO. Total 380 participants developed pre-eclampsia and eclampsia after 12 years follow-up.

**Table 1:** Baseline demographics and exposure of air pollutants in Taiwan.

***N* = 296,784**	***n***	*****%*****
**Age, years**		
<30	119,804	40.4
30–44	123,874	41.8
≥45	53,106	17.8
Mean, (SD)		33.7 (10.4)
**Area**		
North	142,561	48.0
Central	59,082	19.9
Southern	74,028	24.9
Eastern	21,113	7.11
**Urbanization level**		
1 (highest)	99,119	33.4
2	90,742	30.6
3	51,801	17.4
4 (low)	55,122	18.6
**Monthly income**		
<15,000	118,614	40.0
15,000–29,999	140,483	47.3
≥30,000	37,687	12.7
**Occupational class**		
White color class	171,900	57.9
Blue color class	102,413	34.5
Other	22,471	7.57
**Comorbidity**		
Hypertension	53,179	17.9
Diabetes mellitus	46,465	15.6
Chronic kidney disease	3,088	1.04
Obesity	4,879	1.64
Glomrulonephritis	19	0.01
Multiple pregnancy	104,883	35.3
Autoimmune disease	21121	7.12
Nephrotic syndrome	1,542	0.52
Proteinuria	5,003	1.69
Previous preeclampsia	162	0.05
Multiple births	1,856	0.63
**NO**_**x**_ **level (daily average, ppb)**		
Mean, SD^†^		29.2 (12.8)
Min		5.27
Lower quartile		19.8
Median		25.6
Upper quartile		35.9
Max		101
**NO level (daily average, ppb)**		
Mean, SD^†^		9.47 (8.18)
Min		0.84
Lower quartile		4.24
Median		5.98
Upper quartile		12.6
Max		63
**NO**_**2**_ **level (daily average, ppb)**		
Mean, SD^†^		19.7 (5.42)
Min		3.92
Lower quartile		15.6
Median		19.6
Upper quartile		23.6
Max		38
**CO level (daily average, ppm)**		
Mean, SD^†^		0.55 (0.18)
Min		0.11
Lower quartile		0.43
Median		0.51
Upper quartile		0.61
Max		2.29
**Outcome**		
Pre-eclampsia and eclampsia (N, %)		380 (0.13)
Follow-up time, years (mean, SD)		11.9 (0.25)

*NOx, nitrogen oxides; NO, nitrogen monoxide; NO2, nitrogen dioxide; CO, Nitric oxide; PM_2.5_, particle matter with diameter less 2.5 μm; SD, standard deviation*.

*The urbanization level was categorized by the population density of the residential area into 4 levels, with level 1 as the most urbanized and level 4 as the last urbanized*.

*COPD, Chronic obstructive pulmonary disease*.

† * means t test*.

The distribution of urbanization level among different quartile of air pollutant levels was displayed in Table 2. Participants exposed to Q4 level air pollutant including NOx, NO, NO_2_, and CO, most living in the level 1 urbanization areas.

**Table 2:** Baseline urbanization level among quartiles of daily average concentration of air pollutants in Taiwan.

**Air pollutant concentration**	**Quartile 1 (Q1) (lowest)**		**Quartile 2 (Q2)**		**Quartile 3 (Q3)**		**Quartile 4 (Q4) (highest)**		***p*-value**
***N* = 296,784**	***n***	**%**	***n***	*****%*****	***n***	*****%*****	***n***	*****%*****	
**NOx**
**Urbanization level**									<0.0001
1 (highest)	8,967	11.3	3,000	4.4	30,642	41.2	56,510	75.5	
2	22,731	28.6	27,091	39.7	26,736	35.9	14,184	18.9	
3	12,730	16	19,787	29.1	15,141	20.4	4,143	5.53	
4 (lowest)	35,004	44.1	18,237	26.8	1,852	2.49	29	0.04	
**NO**
**Urbanization level**									<0.0001
1 (highest)	8,972	11.2	5,758	8.47	27,896	42.1	56,493	68.5	
2	20,069	25.1	29,547	43.4	20,119	30.4	21,007	25.5	
3	13,764	17.2	18,504	27.2	14,693	22.2	4,840	5.87	
4 (lowest)	37,285	46.5	14,211	20.9	3,458	5.23	168	0.2	
**NO**_**2**_
**Urbanization level**									<0.0001
1 (highest)	7,015	8.86	6,006	8.41	19,318	28.7	66,780	84.6	
2	23,149	29.2	25,294	35.4	35,048	52.1	7,251	9.19	
3	12,282	15.5	22,483	31.5	12,198	18.1	4,838	6.13	
4 (lowest)	36,751	46.4	17,620	24.7	748	1.11	3	0.01	
**CO**
**Urbanization level**									<0.0001
1 (highest)	7,015	8.98	8,449	11.9	38,627	55.6	45028	57.4	
2	21,638	27.7	27,591	39.1	19,564	28.1	21,949	27.9	
3	13378	17.1	17,571	24.9	9,516	13.7	11,336	14.4	
4 (lowest)	36,076	46.2	17,053	24.1	1,808	2.6	185	0.24	

*P-value using chi-square for the comparisons between urbanization level among quartiles of daily average concentration of air pollutants*.

*The urbanization level was categorized by the population density of the residential area into 4 levels, with level 1 as the most urbanized and level 4 as the last urbanized*.

*The daily average air pollutant concentrations were categorized based on quartiles for each air pollutants*.

Table 3 distributions that the incidence rate of pre-eclampsia and eclampsia among levels of all air pollutant concentrations. After controlling for potential confounding factors, subjects in Q1 level of air pollutants as reference group, Q4 air pollutant level exposure increase the risk of pre-eclampsia and eclampsia significantly. For NOx, relative Q1 concentrations, the Q2 (adjusted = 2.20, 95% CI = 1.50–3.22), Q3 (adjusted = 7.28, 95% CI = 4.78), and Q4 (adjusted = 23.7, 95% CI = 13.7–41.1) concentrations were had a significant higher risk of pre-eclampsia and eclampsia. Relative to Q1 NO concentrations, the Q2 (adjusted = 1.82, 95% CI = 1.26–2.63), Q3 (adjusted = 7.53, 95% CI = 5.12–11.0), and Q4 (adjusted = 11.1, 95%CI = 6.72–18.3) concentrations also had a significant higher risk of pre-eclampsia and eclampsia. Relative to Q1 NO_2_ concentration, the Q2 (adjusted = 1.99, 95% CI = 1.37–2.90), Q3 (adjusted = 6.15, 95% CI = 3.95–9.57), and Q4 (adjusted = 32.7, 95% CI = 19.7–54.3) concentrations also had a significant higher risk of pre-eclampsia and eclampsia. People under Q3 and Q4 level exposure were 5.47 and 25.8 times (95% CI = 3.42–8.75 and 95% CI = 16.2–41.2) to develop pre-eclampsia and eclampsia relative to those under Q1 level of CO concentration.

**Table 3:** Difference in pre-eclampsia and eclampsia incidences and associated HRs in participant exposed to various daily average concentration of air pollutants.

**Pollutant levels**	***N***	**Pre-eclampsia**	**PY**	**IR**	**Crude HR**	**Adjusted HR**
		**event**			**(95% CI)**	**(95% CI)**
**NOx (daily average)**
Q1	79,432	50	952,940	0.05	1 (reference)	1 (reference)
Q2	68,115	71	817,036	0.09	1.65 (1.15–2.37)**	2.20 (1.50–3.22)***
Q3	74,371	110	891,725	0.12	2.35 (1.68–3.28)***	7.28 (4.78–11.0)***
Q4	74,866	149	897,374	0.17	3.16 (2.29–4.35)***	23.7 (13.7–41.1)***
**NO (daily average)**
Q1	80,090	60	960,811	0.06	1 (reference)	1 (reference)
Q2	68,020	63	815,908	0.08	1.23 (0.86–1.76)	1.82 (1.26–2.63)**
Q3	66,166	131	793,143	0.17	2.64 (1.94–3.59)***	7.53 (5.12–11.0)***
Q4	82,508	126	989,213	0.13	2.04 (1.50–2.77)***	11.1 (6.72–18.3)***
**NO**_**2**_ **(daily average)**
Q1	79,197	51	950,120	0.05	1 (reference)	1 (reference)
Q2	71,403	71	856,472	0.08	1.54 (1.07–2.21)*	1.99 (1.37–2.90)***
Q3	67,312	84	807,274	0.1	1.93 (1.36–2.74)***	6.15 (3.95–9.57)***
Q4	78,872	174	945,209	0.18	3.42 (2.51–4.68)***	32.7 (19.7–54.3)***
**CO (daily average)**
Q1	78,107	48	937,099	0.05	1 (reference)	1 (reference)
Q2	70,664	41	847,801	0.05	0.94 (0.62–1.43)	1.23 (0.80–1.88)
Q3	69,515	65	833,828	0.08	1.52 (1.04–2.21)*	5.47 (3.42–8.75)***
Q4	78,498	226	940,347	0.24	4.69 (3.43–6.40)***	25.8 (16.2–41.2)***

*Q1: first quartile; Q2: second quartile; Q3: third quartile; Q4: fourth quartile; PY, person-years; IR, incidence rate, per 10,000 person-years; HR, hazard ratio; CI, confidence interval*.

*HR adjusted for age, monthly income, urbanization level, occupational class, and comorbidities*.

The result of stratified Cox regression analysis was shows in Table 4 by considering subject exposed to Q1 level of air pollutants as reference group. Relative to Q1 concentrations of NOx, NO, NO_2_, and CO, the participants who exposed in Q4 level of air pollutants had a significant higher risk to pre-eclampsia and eclampsia in each age group, monthly income, occupational class, and urbanization level 1, 2, 3 and 4. In the Table 5, we found that NOx, NO, NO2, and CO showed significantly positive correlation with each other.

**Table 4:** Incidence rate and hazard ratio of pre-eclampsia and eclampsia between various daily average concentrations of NOx, NO, NO_2_, PM_2.5_, and CO stratified by gender, age, and comorbidities.

	**Adjusted HR (95%CI)**				
**Air pollutants**		**NOx**	**NO**	**NO_2_**	**CO**
**IQR**	**Quartile 1 (lowest)**	**Quartile 4 (highest)**	**Quartile 4 (highest)**	**Quartile 4 (highest)**	**Quartile 4 (highest)**
**Age, years**
<30	1 (reference)	8.54 (4.13–12.7)^***^	8.68 (3.86–19.5)^***^	5.79 (2.57–8.14)^***^	18.5 (8.87–18.7)^***^
30–44	1 (reference)	8.38 (7.97–14.2)^***^	8.22 (3.85–17.5)^***^	3.14 (1.38–7.12)^***^	2.52 (1.19–5.34)^***^
≥45	1 (reference)	5.71 (1.75–18.6)^***^	6.47 (1.51–7.62)^***^	8.00 (2.43–9.62)^***^	4.57 (2.41–10.1)^***^
**Urbanization level**
1 (highest)	1 (reference)	3.14 (1.23–8.03)^*^	1.19 (0.57–2.49)	1.62 (1.54–4.87)^***^	4.04 (1.74–9.38)^**^
2	1 (reference)	7.85 (1.11–9.65)^***^	4.93 (1.22–8.53)^***^	5.42 (2.56–7.35)^***^	11.9 (5.49–25.9)^***^
3	1 (reference)	9.96 (3.39–29.2)^***^	6.09 (5.10–7.37)^***^	5.89 (2.29–15.0)^***^	3.34 (1.97–11.4)^***^
4 (low)	1 (reference)	6.81 (2.81–7.35)^***^	6.53 (3.08–13.6)^***^	2.46 (1.48–11.0)^***^	4.46 (1.62–8.24)^***^
**Monthly income**
<15,000	1 (reference)	12.5 (5.53–28.4)^***^	4.25 (2.01–8.97)^***^	12.1 (5.88–24.9)^***^	12.2 (6.33–23.7)^***^
15,000–29,999	1 (reference)	6.70 (4.85–9.37)^***^	8.53 (3.14–9.61)^***^	9.80 (4.52–12.2)^***^	5.98 (2.84–8.90)^***^
≥30,000	1 (reference)	8.66 (2.10–35.5)^**^	5.00 (1.22–12.3)^***^	5.14 (3.47–16.0)^**^	2.78 (1.34-4.72)^***^
**Occupational class**
White color class	1 (reference)	9.11 (5.44–13.3)^***^	7.46 (3.96–14.0)^***^	2.13 (1.11–4.09)^***^	2.21 (1.19–4.09)^***^
Blue color class	1 (reference)	4.95 (2.45–8.51)^***^	6.99 (2.43–10.2)^***^	14.8 (5.66–18.9)^***^	6.24 (2.56–15.2)^***^
Other	1 (reference)	4.60 (0.77–27.4)	1.56 (0.12–4.59)	13.1 (1.83–19.4)^*^	6.80 (1.61–8.72)^*^*

*Q1: first quartile; Q2: second quartile; Q3: third quartile; Q4: fourth quartile; PY, person-years; IR, incidence rate, per 1,000 person-years; HR, hazard ratio; CI, confidence interval*.

*HR adjusted for age, monthly income, urbanization level, occupational class, and comorbidities*.

* *p < 0.05*;

** *p < 0.01*;

*** *p < 0.001*.

**Table 5:** Description correlation matrix for air pollutants.

	**NOx**	**NO**	**NO_2_**	**CO**
**NOx**	1			
**NO**	0.968^***^	1		
**NO**_2_	0.921^***^	0.799^***^	1	
**CO**	0.811^***^	0.797^***^	0.735^***^	1

*** *correlation is significant at the <0.0001 level (two tails)*.

**Figure 2:**
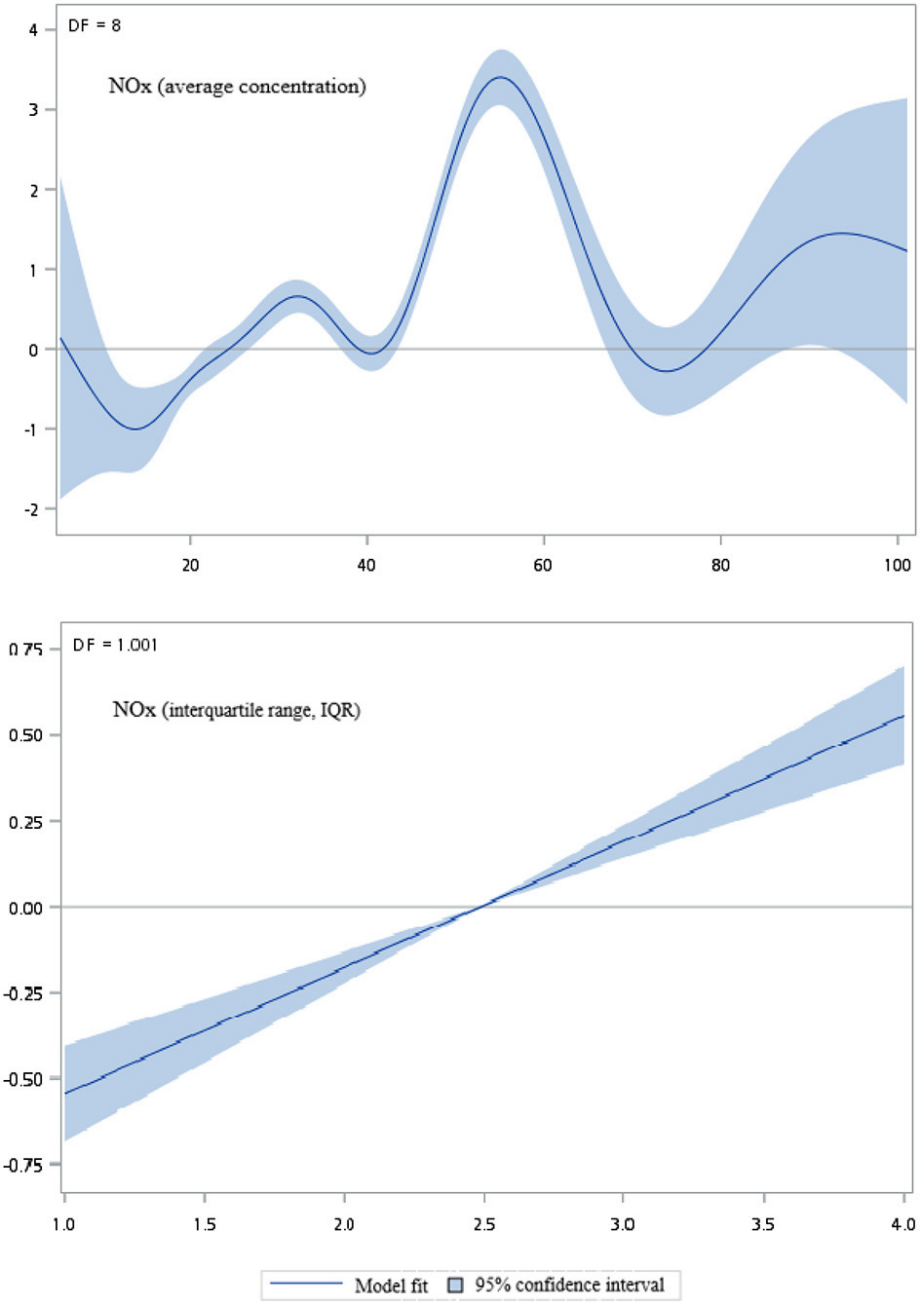
Generalized additive model plot for air pollutions (NOx).

**Figure 3:**
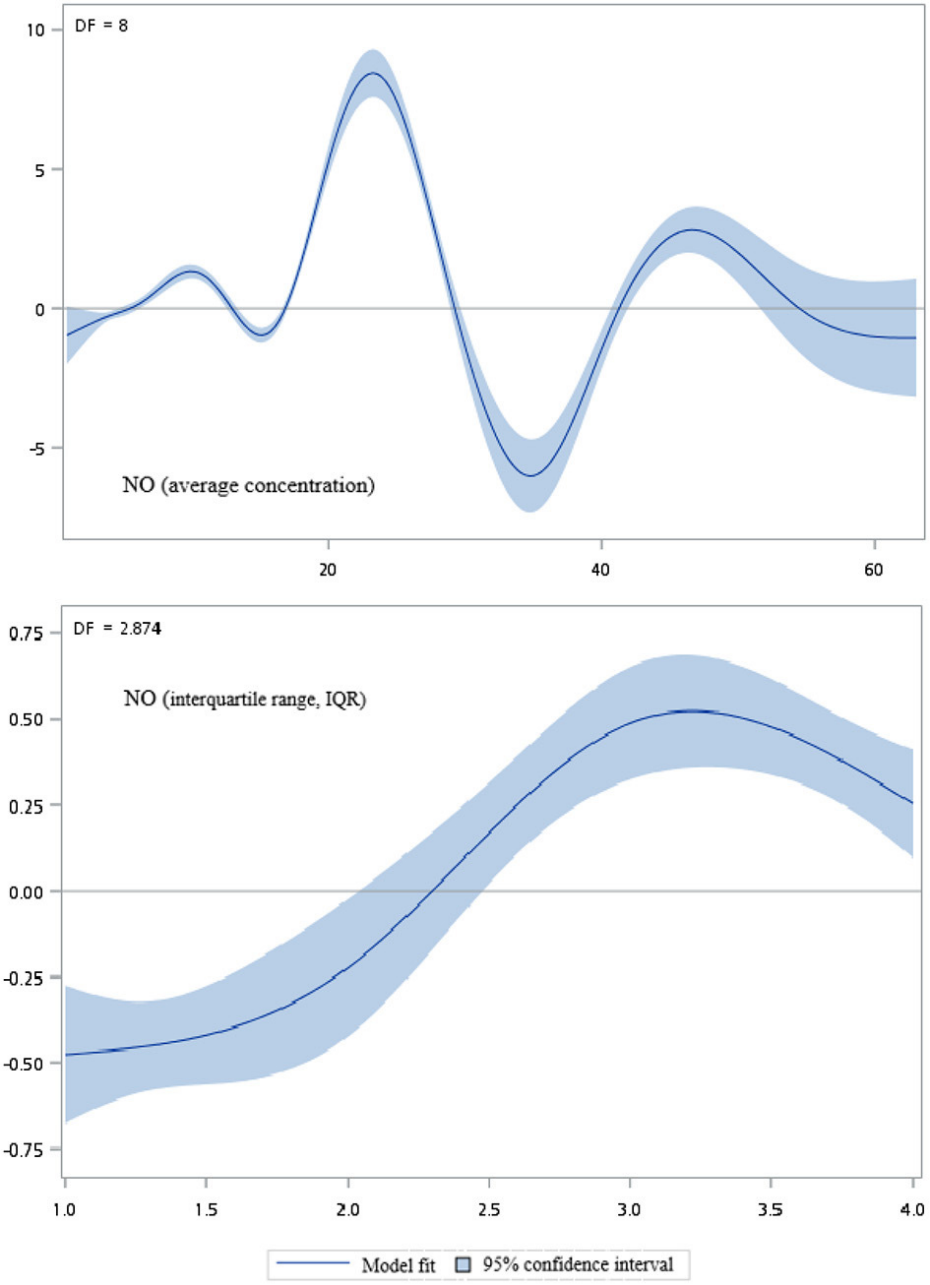
Generalized additive model plot for air pollutions (NO).

**Figure 4:**
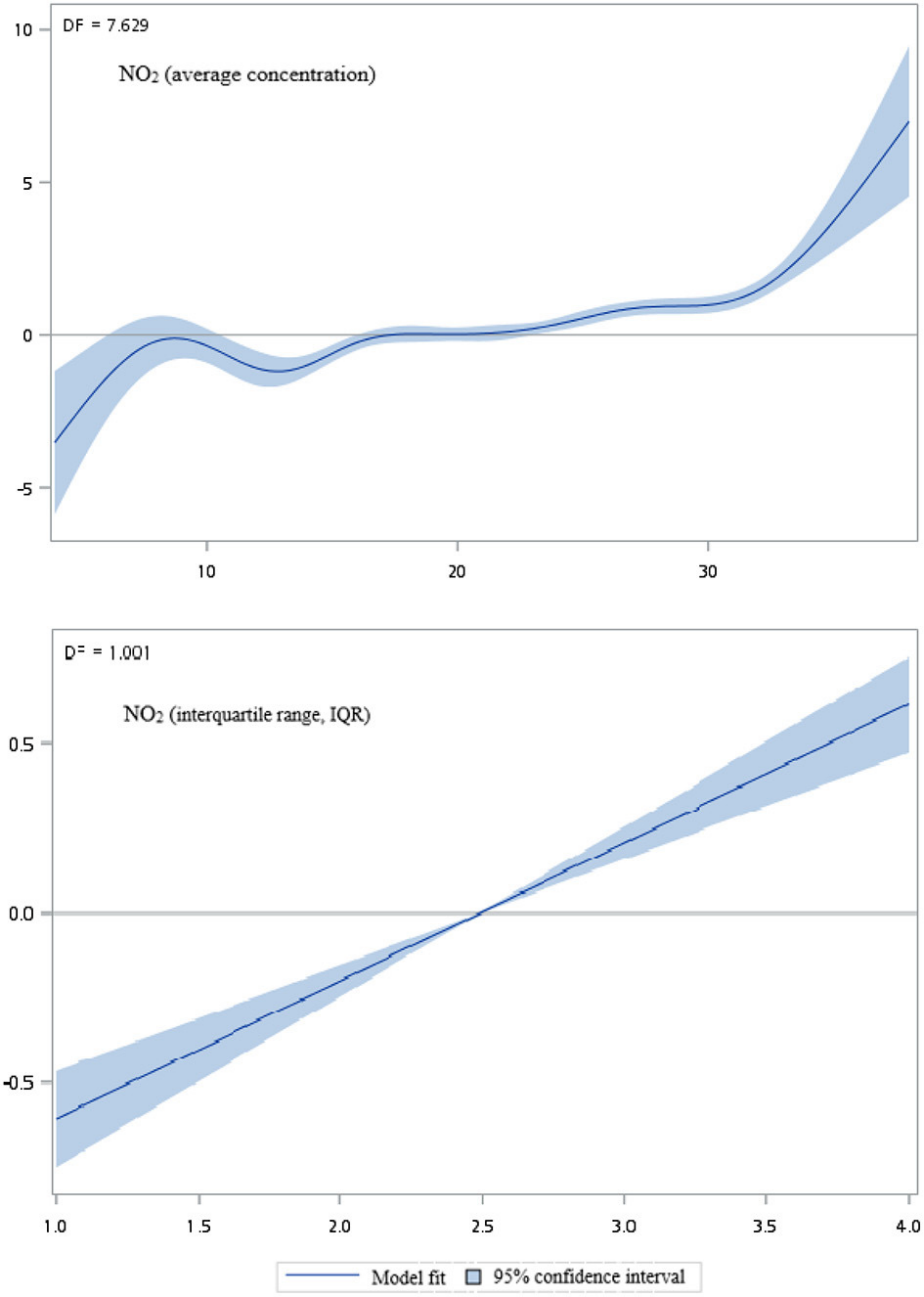
Generalized additive model plot for air pollutions (NO_2_).

**Figure 5:**
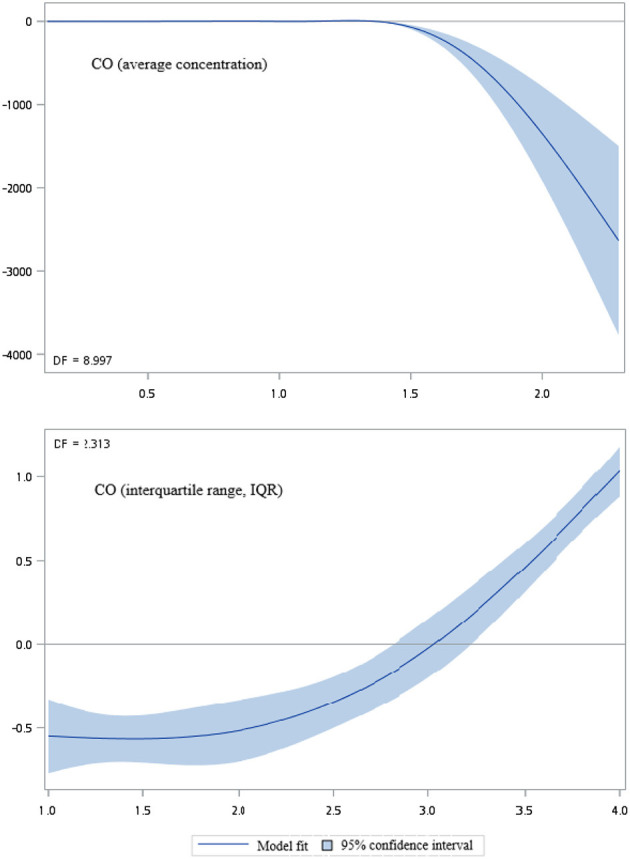
Generalized additive model plot for air pollutions (CO).

Figures 2–5 show that generalized additive model (GAM) was used to quantitatively evaluate the acute effects of air pollution including NOx, NO, NO2, and CO concentrations and interquartile range on the incidence of pre-eclampsia and eclampsia in Taiwan. We found that the plots of the generalized additive model for interquartile range more linear than the plots of generalized additive model for air pollution concentrations.

## Discussion

This study revealed that women exposed to higher concentrations of NO_X_, NO, NO_2_, and CO had higher preeclampsia incidence. Further, we found that extremely high risk of preeclampsia was observable for those exposed to the highest concentrations of NO_X_, NO, NO_2_, and CO, which displayed a dose–response relationship.

It is an interesting finding that just exposure to higher concentrations of particulate air pollutants could be related with adverse pregnancy outcomes, i.e., higher preeclampsia risks. We propose that disturbances of the placenta caused by air pollutants would be a main mechanism underlying the increased incidence of preeclampsia, including hypomethylation or methylation of related genes and increased oxidative stress, vascular indexes, and immune activation (15). To address further, the clinical phenomenon we observed would be attributed to the altered epigenetic regulations of air pollutants during normal placenta development, thus predispose to the development of preeclampsia (31, 32). Lin et al. have pointed out that epigenetic processes include DNA methylation, histone modifications, and non-coding RNA expression (33). Since Janssen et al. and Abrams et al. have found that air pollutants exposure such as NO2 was related to global hypomethylation of DNA as well as methylation of *ADORA2B* of which a gene signify for preeclampsia, epigenetic methylation alterations of placenta caused by air pollutants would be one possible pathway accounting for our findings that exposed to the highest concentrations of nitrogenous compounds was related to higher risks of preeclampsia (34, 35). In addition to methylation, oxidative stress caused by air pollutant is presumed to play a role. Exposure to black carbon and PM_2.5_ have been reported with increased placental nitrosative stress biomarker 3-nitrotyrosine as well as induce hypomethylation of leptin promoters (36, 37). Chikir et al. indicated that placental oxidative and nitrative stress was related to mechanisms in preeclampsia development (38), thus increased oxidative stress of air pollutants exposure during pregnancy could be one explainable pathway why we found that higher risks of preeclampsia were observed in those exposure to highest concentrations of NO_X_, NO, NO_2_, and CO. However, we did not observe similar phenomenon that exposure to highest concentrations of PM_2.5_ was associated with higher risk of preeclampsia as Saenen et al. (37) Further studies would be needed to investigate the clear associations between air pollutants particulates and preeclampsia.

Altered hemodynamics of placenta caused by air pollutants would also be proposed to play a role in development of preeclampsia. It have been reported that exposure to air pollutants causes significant increases in fetal capillary surface area and in total and mass-specific conductance as well as abnormal resistance index, which both could predict preeclampsia risk (39, 40). Because preeclampsia is related to unbalanced blood flow of the placenta, we speculate air pollutants could cause endothelial dysfunction and increased thromboxane activation of the placenta.

Finally, exposure to air pollutants during pregnancy would also our proposed pathway for causing immune activation of the placenta and thus increased risk of preeclampsia. Detmar et al. discovered that embryo exposure to polycyclic aromatic hydrocarbons stimulated uterine natural killer cells into the placenta and increased neonatal loss in their mouse models (15). Since air pollutants hydrocarbon was always accompanied with CO (41), their study might account for our finding that exposure to higher concentrations of CO was associated with higher risk of preeclampsia. Our finding should arouse clinical alertness regarding the effect of heavily air polluted area on maternal health.

This study had several limitations. First, information about body mass index, family history of preeclampsia, ambulatory blood pressure, urine protein, level of blood glucose, level of uric acid, and baseline estimated glomerular filtration rate was unavailable. In addition, nutrition status, *in-vitro* fertilization, and whether it was a multiple pregnancy, all of which are risk factors for preeclampsia, were also unknown. Second, accurate personal exposure to air pollutants was unknown; many pregnant women carry a personal N95 mask, use air cleanser, and avoid heavy traffic whenever they can. Furthermore, although we used postal code to locate the residence of each woman to calculate their air pollutant exposure, a monitoring bias may exist here because workplace, exposure to second-hand smoke, and means of commuting were unknown. Third, we did not analyze at which trimester exposure to air pollutants for risk of preeclampsia would be the highest.

## Conclusion

This study demonstrated that exposure to air pollutants NO_X_, NO, NO_2_, and CO are associated with increased preeclampsia risk. For maternal health, additional studies confirming the association between preeclampsia and air pollutant exposure and investigating the involved mechanism and protective methods for maternal and fetal well-being are warranted.

## Data Availability Statement

The dataset used in this study is held by the Taiwan Ministry of Health and Welfare (MOHW). The Ministry of Health and Welfare must approve our application to access this data. Any researcher interested in accessing this dataset can submit an application form to the Ministry of Health and Welfare requesting access. Please contact the staff of MOHW (Email: stcarolwu@mohw.gov.tw) for further assistance. Taiwan Ministry of Health and Welfare Address: No.488, Sec. 6, Zhongxiao E. Rd., Nangang Dist., Taipei City 115, Taiwan (R.O.C.). Phone: +886-2-8590-6848. All relevant data are within the paper. Requests to access the datasets should be directed to stcarolwu@mohw.gov.tw.

## Ethics Statement

The studies involving human participants were reviewed and approved by This study was approved to fulfill the condition for exemption by the Institutional Review Board (IRB) of China Medical University (CMUH-104-REC2-115-AR4). The IRB also specifically waived the consent requirement. Written informed consent for participation was not required for this study in accordance with the national legislation and the institutional requirements.

## Author Contributions

S-YL and C-HK: conception/design. C-HK: provision of study materials. All authors: collection and/or assembly of data, data analysis and interpretation, manuscript writing, and final approval of manuscript.

## Conflict of Interest

The authors declare that the research was conducted in the absence of any commercial or financial relationships that could be construed as a potential conflict of interest.

## Supplementary Material

Supplementary Figure 1 Site map of air quality monitoring stations in Taiwan.
