# Supplementary figures and images for: Association Between Preeclampsia Risk and Fine Air Pollutants and Acidic Gases: A Cohort Analysis in Taiwan
Source: Front Public Health. 2021 Mar 31;9:617521. doi: 10.3389/fpubh.2021.617521 (PMC8044398; doi:10.3389/fpubh.2021.617521)

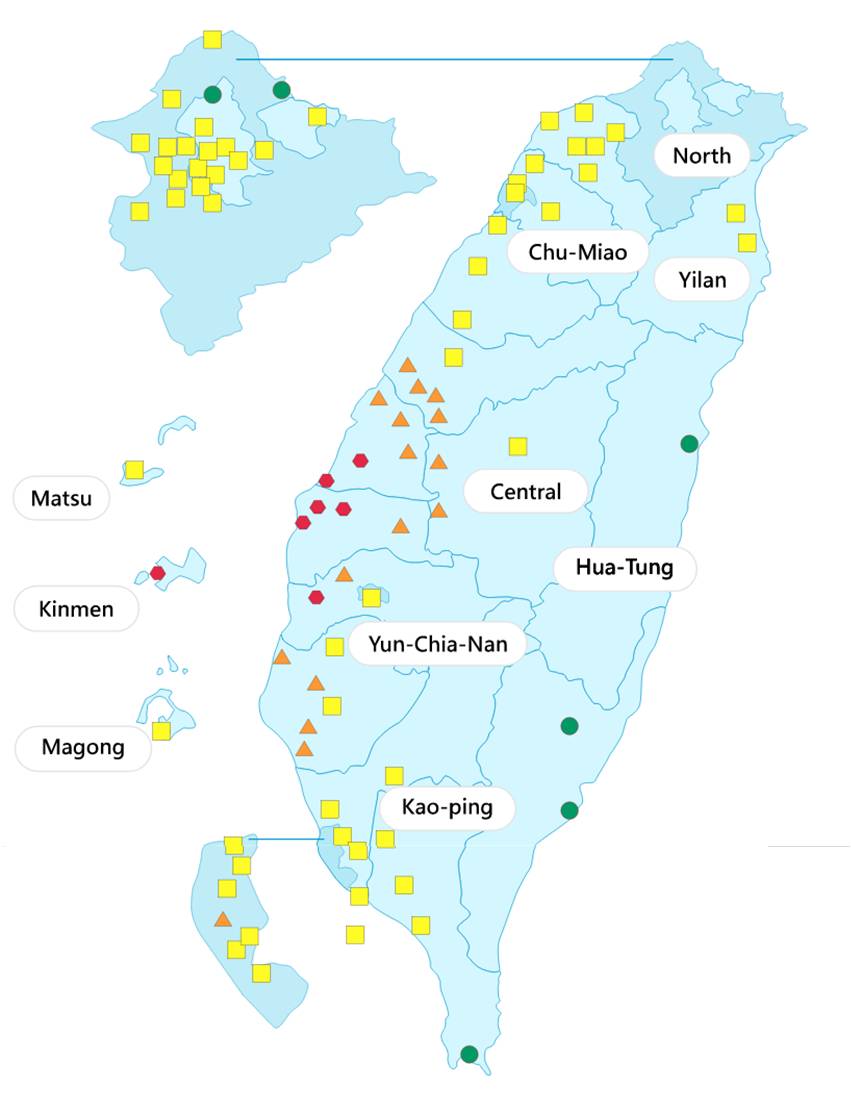

Supplement: Supplementary Figure 1 — Site map of air quality monitoring stations in Taiwan. [file Image_1.jpg]
